# Extracellular calcium promotes internalization and degradation of the fission yeast TRP-like calcium ion channel Pkd2

**DOI:** 10.17912/micropub.biology.001265

**Published:** 2024-08-05

**Authors:** Takayuki Koyano, Kaori Onishi, Makoto Matsuyama, Masaki Fukushima, Kazunori Kume

**Affiliations:** 1 Division of Cell Biology, Shigei Medical Research Institute, Okayama, Okayama, Japan; 2 Division of Molecular Genetics, Shigei Medical Research Institute, Okayama, Okayama, Japan; 3 Shigei Medical Research Hospital, Okayama, Okayama, Japan; 4 Graduate School of Integrated Sciences for Life, Hiroshima University, Higashi-Hiroshima, Hiroshima, Japan; 5 Hiroshima Research Center for Healthy Aging (HiHA), Hiroshima University, Higashi-Hiroshima, Hiroshima, Japan

## Abstract

The correct localization of proteins is linked to their cellular function. The *Schizosaccharomyces pombe* Pkd2 localizes to the endoplasmic reticulum and plasma membrane. Here we investigate the behavior of Pkd2 in response to calcium. Pkd2-GFP, normally enriched at the cell ends, is reduced from the plasma membrane by CaCl _2_ addition, while cytoplasmic dots and free GFP are increased. This suggests that Pkd2 is internalized and degraded in response to extracellular CaCl _2_ . This internalization is partially suppressed by treatment with an Arp2/3 inhibitor, CK-666. Our data provide new insights into the relationship between Pkd2 internalization and calcium response.

**Figure 1. The behavior of fission yeast Pkd2 in response to external CaCl 2:**
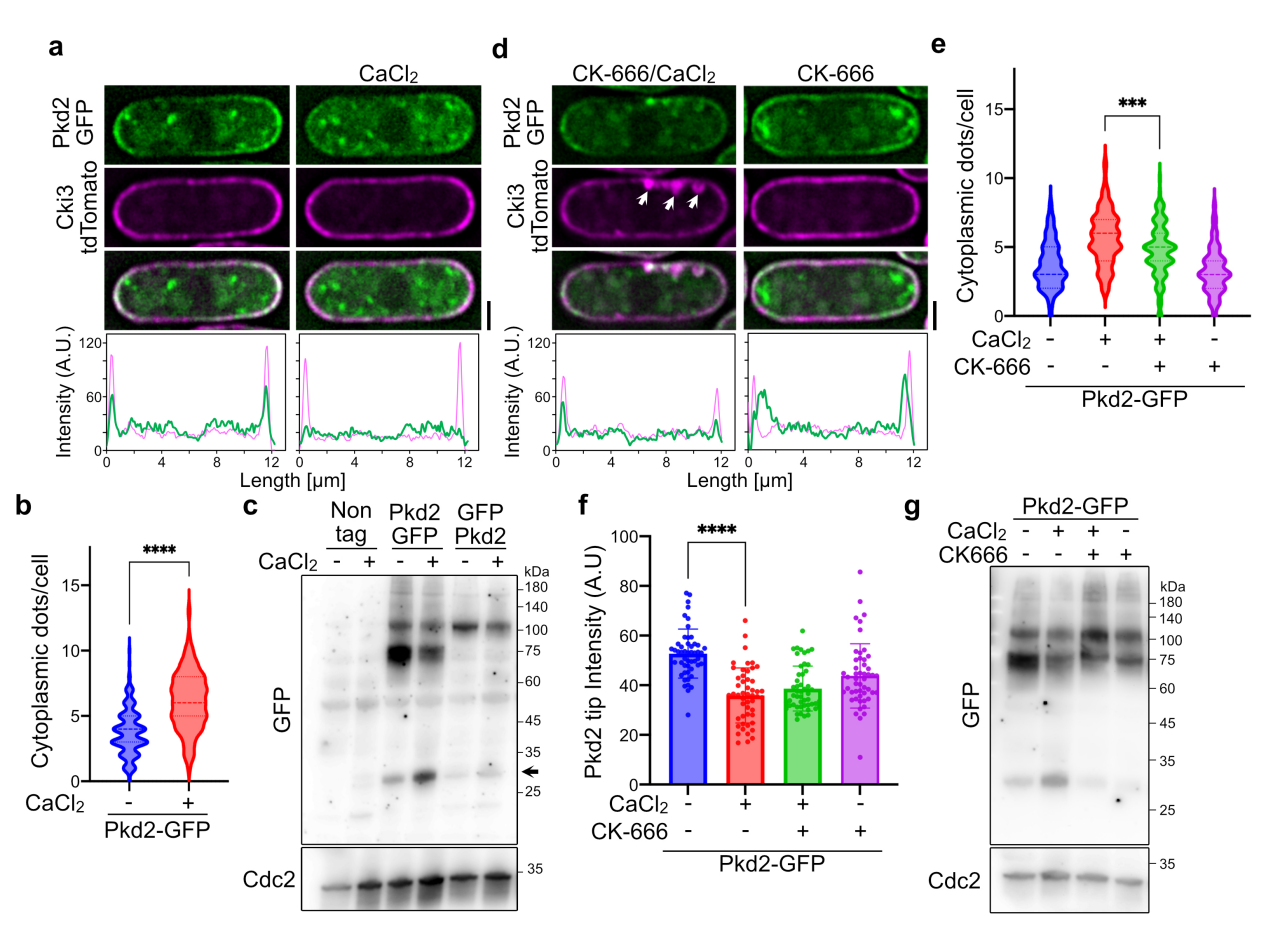
(a) Representative images of Pkd2-GFP in the absence or presence of an additional 0.2M CaCl _2_ . The plasma membranes were marked by Cki3-tdTomato. The line plots were carried out along with the long axis of the cells. Cki3 peaks indicate the tips of the cells. Bar, 2 µm. (b) The number of Pkd2-GFP cytoplasmic dots per cell (n > 100). Cells were cultured in the absence or presence of an additional 0.2M CaCl _2_ for 2 h. Dashed lines and dotted lines in the violin plot indicate median and quartiles, respectively. (c) Western blotting analysis. Whole-cell extracts were prepared from the indicated strains in the absence or presence of an additional 0.2M CaCl _2_ and immunoblotting was carried out with anti-GFP and anti-Cdc2 (as a control) antibodies. The positions of size markers are shown on the right. Arrow indicates the position of GFP size. (d) Representative images of Pkd2-GFP. Cells were cultured for 2 h in the absence or presence of 0.2 M CaCl _2_ and 200 µM of CK-666. Arrows indicate the abnormal Cki3 localization patterns. Bar, 2 µm. (e) The number of Pkd2-GFP cytoplasmic dots in the indicated culture conditions (n > 50). (f) The intensity of Pkd2-GFP at the cell tips (n = 50). (g) Western blotting analysis. Whole-cell extracts were prepared from the indicated culture conditions and immunoblotted with anti-GFP and anti-Cdc2 (as a control) antibodies. All p values were obtained from the two-tailed unpaired Student's t test. ****p < 0.0001, ***p = 0.0002.

## Description

Mutations in *PKD2* gene cause autosomal dominant polycystic kidney disease (ADPKD), which is one of the most frequent genetic kidney diseases (Cornec-Le Gall et al., 2019; Mochizuki et al., 1996) . *PKD2* encodes Polycystin-2 ( Pkd2 ), a cation channel in the primary cilium membrane and endoplasmic reticulum (ER) of renal collecting duct cells (Ma et al., 2017; Padhy et al., 2022) . Pkd2 also has an essential role in determination of left-right symmetry in mouse embryos (Yoshiba et al., 2012) . Pkd2 preferentially localizes to the dorsal side of a cilium membrane to sense the direction of nodal flow (Katoh et al., 2023) . However, little is known how these spatial localizations are regulated.

Fission yeast Pkd2 shares some similarities but does not complement human Pkd2 (h Pkd2 ) (Koyano et al., 2023; Malla et al., 2023) . Fission yeast Pkd2 also localizes to both the ER and plasma membrane, like mammalian Pkd2 (Koyano et al., 2023) . The N-terminal region including a signal sequence of Pkd2 and 9 transmembrane domains are required for ER localization (Koyano et al., 2023; Malla et al., 2023) . Depletion of the C-terminal region of Pkd2 enhances eisosomal localization and suppresses internalization (Malla et al., 2023) . Although Pkd2 internalization and degradation have been reported (Aydar & Palmer, 2009; Malla et al., 2023) , the details are still unknown.

We first checked the cellular localization by a fluorescence microscope. C-terminally GFP-tagged Pkd2 (Pkd2-GFP) localized to the plasma membrane, marked by Cki3 (Koyano et al., 2015) , and cytoplasm as a dot; however, plasma membrane localization was attenuated and the cytoplasmic dots were increased in the externally CaCl _2_ added condition ( Figure 1a ). Line plots indicated that Pkd2-GFP signals peaked at both cell ends where Cki3 also peaked ( Figure 1a ). On the other hand, GFP signals were decreased from the plasma membrane and cytoplasmic dots were increased in the externally CaCl _2_ added condition ( Figure 1a, b ). These data indicate that Pkd2 internalization is induced by extracellular calcium.

We have previously shown that Western blotting analysis shows different band patterns depending on the position of GFP tagging (Koyano et al., 2023) . We checked whether Pkd2 protein behaviors are affected by extracellularly added calcium, as Pkd2 is involved in calcium influx and calcineurin-dependent signaling pathways (Koyano et al., 2023; Ma et al., 2011; Poddar et al., 2022) . Consistent with previous data, N-terminally GFP-tagged Pkd2 (GFP- Pkd2 ) showed a single full-length band that was slightly decreased by extra CaCl _2_ ( Figure 1c ). On the other hand, the extract from Pkd2-GFP expressing cells showed 2 major bands, a full-length sized band (~110kDa) and a cleaved-sized band (~75kDa) by the Western blotting analysis ( Figure 1c ). In addition to 2 major bands, there was a weak band around GFP size (~28kDa) ( Figure 1c ). Interestingly, the signal of the GFP band increased with the addition of CaCl _2_ , whereas the signals of the 2 bands, especially the cleaved band (~75kDa) decreased ( Figure 1c ). The free GFP signal was taught to be enhanced by the Pkd2 degradation since Pkd2 reportedly localizes to the vacuole and is degraded (Malla et al., 2023) . Taken together, we propose that Pkd2 is internalized and subsequently degraded in response to the external calcium.

The previous report suggests that endocytosis is involved in Pkd2 internalization process (Malla et al., 2023) . In fission yeast, Arp2/3 plays a critical role in clathrin-mediated endocytosis (Galletta & Cooper, 2009; Marek et al., 2020) . We then examined the effect of CK-666, an Arp2/3 specific inhibitor (Nolen et al., 2009) , on Pkd2 internalization. The cytoplasmic Pkd2-GFP dots disappeared with the treatment of CK-666 ( Figure 1d, e ); however, membrane intensities at the cell tips were not fully recovered ( Figure 1d, f ). It is noted that Pkd2 and Cki3 showed abnormal localization patterns in the double treatment condition of CaCl _2_ and CK-666 ( Figure 1d, arrows ). Concomitantly, the GFP band vanished from the gel by treatment with CK-666 in both the presence and absence of extra CaCl _2_ ( Figure 1g ). We conclude that Pkd2 internalization and subsequent degradation in response to extracellular calcium is partially promoted by Arp2/3-dependent endocytosis. Further analysis will reveal the biological significance of Pkd2 internalization and degradation in response to calcium.

## Methods

**Yeast method**

Standard media and methods for fission yeast were used (Moreno et al., 1991) . Strains used in this study are listed in the Reagents section. The strains were grown in YE5S media and incubated at 27°C. For CaCl _2_ treatment, 1 mL of 2M CaCl2 was added to 9 mL of the overnight culture (OD600: 0.3-0.6) and cultured for an additional 2 h. 20 mM CK-666 (Sigma-Aldrich, SML0006) was prepared in DMSO and stored at -20°C until use. 100 µL of 20 mM CK-666 is added to the 10 mL cell culture (final concentration: 200 µM).

**Microscopy**

Fluorescence microscope images were obtained by the Olympus IX83 inverted microscope system with UPLXAPO 60x objective lens (NA 1.42, immersion oil) and a DP80 digital camera. The cells were collected by the centrifuge at 5,000rpm for 1 min, and spotted onto a glass slide (Matsunami glass). The cells were observed immediately after covering with a coverslip. Deconvolved images were shown in Figures. The signal intensities were measured by using Image J (Line Plot Profile). Pkd2-GFP intensities at the cell tips were obtained from where Cki3-tdTomato intensities were peak. Images were processed by using CellSens Dimension (Evident) and affinity photo 2.

**Western blotting**

Whole-cell extracts were prepared based on the alkaline method (Matsuo et al., 2006) and as described previously (Koyano et al., 2023) . The samples were separated by 10% of SDS-PAGE gel (Bio-rad, 4561035) and transfer to a PVDF membrane. The membranes were blocked with 5 % of skim milk in TBS-tween20 (TBST) for 30 min at room temperature, subsequently incubated with Anti-GFP (Roche, 11814460001) at 4°C overnight. After washing with TBST, the membranes were incubated with anti-Mouse (Thermo Fisher Scientific, G-21040) at room temperature for 60 min. To efficiently detect the GFP signal, Can Get Signal ^TM^ immunoreaction enhancer solution (TOYOBO, NKB101) was used. Then the membranes were incubated with Western Blot Quant HRP substrate (Takara Bio, T7102). For the control, the membranes were re-incubated with anti- Cdc2 (SantaCruz Biotechnology, SC-53217) in TBST with 0.1% of sodium azide at room temperature for 3 h. Amersham Image Quant 800 (Cytiva) was used for detection of chemiluminescence.

## Reagents

The strains used in this study and their genotypes are listed below.

**Table:** 

Strain	Genotype	Reference
513	*h- leu1-32 ura4-D18*	Lab stock
TK1323-1	* h- Δ pkd2 ::kanMX leu1-32:P _pkd2_ -GFP- pkd2 ^+^ -T _pkd2_ -leu1 ^+^*	Koyano et al., 2023
UKK2767	* h- pkd2 ^+^ :GFP:hphMX *	This study
TK1818-2	* h- pkd2 ^+^ :GFP:hphMX cki3 ^+^ :tdTomato:kanMX leu1-32 *	This study
